# Beyond the single biopsy: Sampling dynamic disease ecosystems for patient stratification and translational validation

**DOI:** 10.1002/ctm2.70809

**Published:** 2026-09-08

**Authors:** Kangjie Shen, Xin Huang, Tao Zan

**Affiliations:** ^1^ Department of Plastic and Reconstructive Surgery Shanghai Ninth People's Hospital Shanghai Jiao Tong University School of Medicine Shanghai China

1

Dear Editor,

Precision medicine often treats a biopsy as a faithful miniature of the disease from which it was taken. However, cell states, molecular programs and therapeutic vulnerabilities vary across anatomical compartments, clinical states and treatment phases. A biopsy therefore records one spatial coordinate, one disease state and one treatment time point. Single‐cell RNA sequencing can resolve cellular composition, while spatial methods retain the tissue coordinates needed to interpret it, but both remain dependent on how the specimen was selected and annotated. Sampling context is part of the biological measurement: errors at this step can propagate into patient stratification and the validation of biomarkers or therapeutic targets.

Cancer established the spatial dimension of this problem. Multiregion sequencing of renal carcinomas showed that most somatic alterations were absent from at least one sampled region and that favourable and unfavourable prognostic signatures could coexist within the same tumour.^1^ In head and neck cancer, single‐cell analysis of primary and metastatic tumours, followed by in situ validation, localised partial epithelial‐to‐mesenchymal‐transition states to the leading edge of primary tumours.^2^ A central biopsy can thus recover abundant malignant cells while missing the interface where invasive, stromal and immune programs are organised. The relevant question shifts from whether a marker is present to where it is present and which cells produce it.

Treatment makes the timing of sampling clinically consequential. Pretreatment specimens may support outcome prediction, whereas serial biopsies are required to capture treatment‐induced change. In melanoma, a spatially informed signature derived from pretreatment specimens predicted immunotherapy outcome in an independent validation cohort and outperformed published bulk‐expression signatures.^3^ Its predictive meaning was therefore anchored to the pretreatment specimen. In triple‐negative breast cancer, serial biopsies obtained at baseline, after pembrolizumab and after pembrolizumab plus radiotherapy revealed three response trajectories by single‐cell transcriptomics and spatial proteomics.^4^ One responder group was already immune active at baseline, whereas another developed its strongest immune response only after combination therapy; non‐responders showed little immune change. Prediction and response monitoring therefore require different sampling windows. Baseline tissue captures the state from which response begins, on‐treatment tissue captures adaptation, and residual or recurrent tissue captures persistence and escape. The same molecular signal may change in abundance, cellular source or location across these phases.

The implications of spatiotemporal heterogeneity for sampling are not confined to cancer. Keloids provide a complementary example because depth, radial position and clinical activity can be directly annotated. Integrated single‐cell and spatial transcriptomics localised disease‐associated fibroblast states to deep lesional regions near endothelial‐transcript‐rich spots.^5^ Activity and radial position added another layer: pro‐inflammatory fibroblasts were more abundant in active than inactive lesions, angiogenic tip and immature endothelial cells were enriched at the active periphery, and endothelial mesenchymal activation was most prominent in the active centre.^6^ Predefined‐region single‐cell RNA sequencing distinguished the hypercellular zone, dominated by extracellular‐matrix‐producing myofibroblasts, from the infiltrating margin, enriched in mononuclear phagocytes and endothelial cells.^7^ Paired biopsies from 48 human keloids, obtained before and after intralesional treatment with 5‐fluorouracil or triamcinolone acetonide, then added a treatment dimension: mature vessels and lymphatics increased specifically in the middle dermis of responding lesions, whereas pretreatment hypoxia and vascular measures did not distinguish subsequent responders from non‐responders.^8^ Thus, depth, radial position, activity and treatment phase shape which fibrovascular and immune programs enter the molecular readout (Figure 1).

**FIGURE 1:**
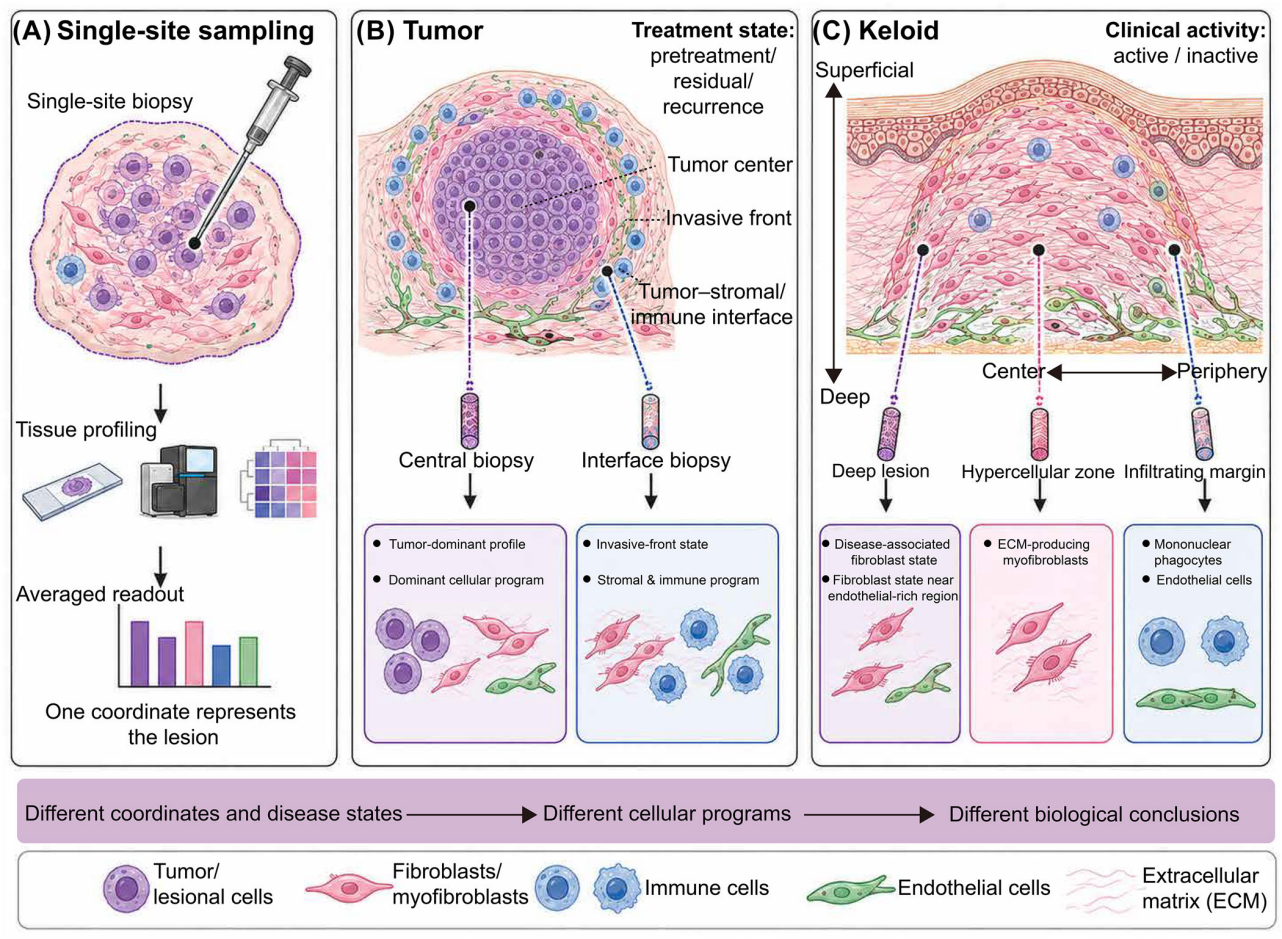
A biopsy samples a coordinate within a dynamic disease ecosystem. Single‐site sampling produces an averaged molecular readout from one spatial coordinate. In tumours, the centre, invasive front and tumour‒stromal/immune interface can contain different cellular programs. In keloids, sampling depth, centre‒periphery position, hypercellular and infiltrating zones, and clinical activity likewise affect the states recovered. Different sampling coordinates may therefore support different biological interpretations.

Neural organisation adds another spatial dimension to sampling. In human keloids, single‐cell analysis with tissue validation showed that asporin (ASPN)‐positive fibroblasts clustered around nerve fibres and lay closer to IGFBP5‐positive Schwann cells. Midkine (MDK) was upregulated in this fibroblast population, and its tissue expression correlated with pain and pruritus.^9^ A complementary study localised abnormally dense catecholaminergic neurites to the reticular dermis of both keloid centres and borders and linked adrenergic signalling to an osteogenic, matrix‐producing fibroblast program.^10^ Together, these studies show that neural–stromal programs in keloids are spatially organised rather than uniformly distributed; their interpretation therefore requires reticular depth and nerve proximity to be recorded alongside centre–border position.

These observations define a practical sequence for translational study design. First, the clinical question should determine the sampling coordinate and treatment phase. Tumour protocols should distinguish the centre, invasive front, stromal or immune interface, and metastatic site, together with pretreatment, on‐treatment, residual or recurrent status. Keloid protocols should record centre–periphery position, superficial–deep level, hypercellular or infiltrating zone, activity status, previous treatment, recurrence, sensory symptoms and relevant vascular or neural context. Paired multiregion sampling can separate regional variation from between‐patient variation, while serial sampling can resolve change within the same patient or lesion. The unit of comparison should be prespecified as region within a lesion, change within a patient, or difference between patients. Second, coordinate‐annotated profiles should be assembled into spatially and temporally defined disease states for patient stratification. Compartment‐specific programs discovered by single‐cell or spatial platforms can then be reduced to scalable in situ assays that preserve location and cellular source. Third, translational validation should test whether a candidate remains informative across regions, patients and clinically meaningful disease phases, localises to the intended niche, and withstands orthogonal spatial and functional assessment. Signals confined to one region or transient state define context‐specific biomarkers or therapeutic targets, with an indication tied to the state in which they occur. This ordering prevents coordinate effects from being misread as stable patient traits (Figure 2).

**FIGURE 2:**
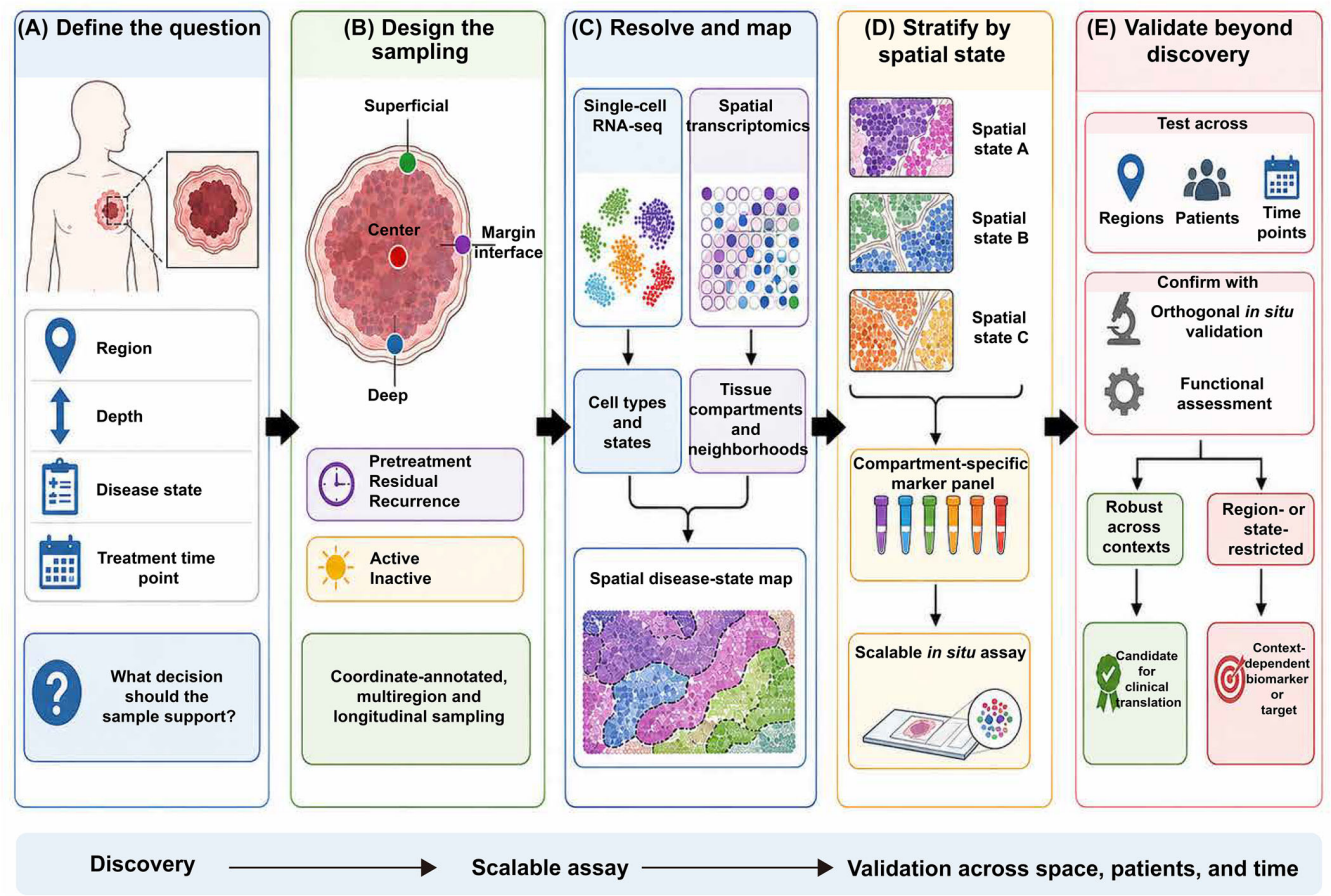
A spatially informed pathway from sampling to clinical translation. The clinical question first defines the relevant spatial and temporal coordinates. Single‐cell RNA sequencing resolves cell types and states, whereas spatial transcriptomics maps them to tissue compartments and neighbourhoods. Integrated disease‐state maps can support patient stratification and the derivation of compartment‐specific marker panels for scalable in situ testing. Candidate biomarkers and therapeutic targets should then be validated across regions, patients and clinically relevant time points using orthogonal spatial and functional approaches.

For keloids, longitudinal sampling across growth, treatment and recurrence remains a major unmet need. More broadly, incorporating each biopsy's spatial and temporal context into patient stratification and validation can strengthen the clinical interpretability and translational value of spatial discoveries.

## CONFLICT OF INTEREST STATEMENT

The authors declare they have no conflicts of interest.

## ETHICS STATEMENT

Not applicable.

## Data Availability

Data sharing is not applicable to this article because no new data were created or analysed in this study.
